# ISDE guidelines on the management of cT2N0 esophageal cancer

**DOI:** 10.1093/dote/doag019

**Published:** 2026-03-05

**Authors:** Geoffrey P Kohn, Virginia Litle, Yousif Eliya, Samantha Leng, Mohammad Asghari-Jafarabadi, Nicolas Contreras, Andrew Davies, Rudy Lackner, Kimberley S Mak, Tom Mala, Ben Markman, Sarbajit Mukherjee, Christopher Nevala-Plagemann, Elizabeth Smyth, Javed Sultan, Stephanie Worrell, Shun Yamamoto, Bas P L Wijnhoven, Ewen A Griffiths

**Affiliations:** Department of Surgery, Monash University, Melbourne, Vic, Australia; Department of Surgery, Cabrini Health, Melbourne, Vic, Australia; Sutter Health, San Francisco, CA, USA; Department of Surgery, Cabrini Health, Melbourne, Vic, Australia; Department of Surgery, Eastern Health, Melbourne, Vic, Australia; Cabrini Research, Cabrini Health, Malvern, Vic 3144, Australia; School of Public Health and Preventive Medicine, Monash University, Melbourne, Vic, Australia; Department of Psychiatry, School of Clinical Sciences, Monash University, Clayton, Vic, Australia; Department of Surgery, University of Utah, Salt Lake City, UT, USA; Department of Surgery, Guy’s and St Thomas’ National Health Service Foundation Trust, London, UK; Department of Surgery, Section of Thoracic Surgery, University of Nebraska, Omaha, NE, USA; Department of Radiation Oncology, Boston Medical Center, Boston, MA, USA; Section of Upper Gastrointestinal Surgery, Oslo University Hospital/University of Oslo, Oslo, Norway; Department of Surgery, Monash University, Melbourne, Vic, Australia; Alfred Health, Melbourne, Vic, Australia; Miami Cancer Institute, Baptist Health South Florida, Miami, FL, USA; Department of Oncology, Huntsman Cancer Institute, University of Utah, Salt Lake City, UT, USA; Department of Oncology, Oxford University Hospitals National Health Service Foundation Trust, Oxford, UK; Salford Royal Hospital, Northern Care Alliance National Health Service Foundation Trust, Salford, UK; Division of Cancer Sciences, School of Medical Sciences, University of Manchester; Department of Surgery, Section of Thoracic Surgery, University of Arizona, Tucson, AZ, USA; Department of Head and Neck, Esophageal Medical Oncology, National Cancer Center Hospital, Tokyo, Japan; Department of Surgery, Erasmus University Medical Center, Rotterdam, The Netherlands; Department of Upper Gastrointestinal Surgery, University Hospitals Birmingham National Health Service Foundation Trust, Birmingham, UK; Institute of Immunotherapy and Immunology, University of Birmingham, UK

**Keywords:** clinical practice guidelines, esophageal neoplasms, esophagectomy, neoadjuvant therapy, neoplasm staging

## Abstract

Esophageal cancer incidence is rising globally, with at least 500,000 new cases diagnosed annually. Management options for non-metastatic disease include primary resection, neoadjuvant or perioperative therapies, or definitive non-surgical treatment, with the choice being guided by tumor staging, histology, patient fitness, and available resources. However, even with the use of advanced diagnostic modalities, preoperative clinical staging is challenging with respect to accuracy of both tumor and nodal assessment. Early-stage esophageal cancer may be managed with local therapies, such as endoscopic mucosal resection or submucosal dissection, while for more advanced tumors managed with curative intent neoadjuvant oncologic therapy is commonly recommended. However, between these two groups lies an infrequent but important subgroup of patients, clinically staged cT2N0M0 esophageal cancer. Guidelines such as the NIH’s National Cancer Institute recommends either surgery alone or neoadjuvant therapy followed by surgery for AJCC Stage I cancers, and add the option of definitive chemoradiation for Stage II disease. With cT2N0 disease straddling both AJCC classifications, management guidance is lacking. This guideline will provide an evidence-based recommendation from the International Society For Disease Of The Esophagus on the management of cT2N0 esophageal cancer, of all types. The recommendations are intended to support surgeons, oncologists, and patients in decisions about the best practice preoperative oncologic management of cT2N0M0 esophageal cancer.

A Working Group within the International Society for Diseases of the Esophagus (ISDE) Guidelines Committee performed a systematic review of the literature. Results of the systematic review were presented to a panel of experts and these results informed the panel discussion about the guideline. This panel used Grading of Recommendations Assessment, Development, and Evaluation approach to deliberate and formulate recommendations.

The panel agreed on a conditional recommendation for the use of neoadjuvant therapy followed by surgery over primary surgical resection (PSR) for adult patients with cT2N0M0 esophageal cancer.

Preoperative clinical staging of esophageal cancer is uncertain, with deficiencies in all diagnostic modalities. However, when all modern staging techniques are utilized, the ISDE recommends neoadjuvant therapy followed by surgical resection as the favored treatment of cT2N0 esophageal cancer. Certain patient groups may still be offered PSR, particularly those unable to tolerate neoadjuvant therapies, or those patients with very low risk of lymph node metastasis as suggested by histological features, small tumor size, and other features.

## Interpretation of strong and conditional recommendations

Guideline recommendations are typically classified as a ‘strong’ or ‘conditional’ recommendation. The statement ‘the guideline panel recommends’ are used for strong recommendations, and ‘the guideline panel suggests’ for conditional recommendations, as per the Grading of Recommendations Assessment, Development, and Evaluation approach.^3^ A strong recommendation signals that almost all clinicians should follow the recommended course of action, with the best available evidence suggesting desirable effects clearly outweigh undesirable effects. A conditional recommendation signals that the benefits of adhering to a recommendation probably outweigh the harms although it does also indicate uncertainty. This uncertainty may be due to a lack of high-quality evidence or variability in how individual patients value the outcomes of interest.

## How to use these guidelines

These guidelines are primarily intended to aid physicians make decisions about management of patients preoperatively diagnosed with cT2N0M0 esophageal cancer of all histological types. They are also intended to educate, inform policy and advocacy, and to identify future research needs. Clinical decision making is multifaceted, and these guidelines are intended to suggest, but not mandate, an acceptable approach to management of this disorder. Finally, these guidelines can also be used by patients as a basis of discussion with their treating physicians.

## Key question addressed by these guidelines

Should PSR vs. neoadjuvant therapy followed by surgery be used for cT2N0 esophageal cancer in adults?

## Recommendations

**Should PSR vs. neoadjuvant therapy followed by surgery be used for cT2N0 esophageal cancer in adults?**

Given the limitations of staging accuracy and the absence of direct trial data, the ISDE Guideline panel suggests that NEOADJUVANT THERAPY FOLLOWED BY SURGERY IS PREFERABLE TO PSR FOR MOST PATIENTS WITH cT2N0 ESOPHAGEAL CANCER. *(Conditional recommendation*, *low certainty of evidence.)*

Remark: primary surgery alone may be considered in exceptional cases meeting all of the following: small, well-differentiated tumor, no LVI, and high confidence in staging workup.

No separate recommendation is made by histological subtype, as the available evidence was insufficient to show differential effects.

The evidence does not demonstrate a survival advantage for neoadjuvant therapy in cT2N0 esophageal cancer. However, the panel’s consensus was that high rates of nodal upstaging would favor the use of neoadjuvant therapy and avoid under-treatment of patients in real-world practice.

## INTRODUCTION

### Aim of these guidelines and specific objectives

The purpose of these guidelines is to provide recommendations based on available evidence and expert opinions regarding the management of all histological types of esophageal cancer staged clinically as cT2N0 by AJCC 8th edition. The target audience includes patients, surgeons, and oncologists. Policy makers and insurance providers interested in these guidelines include those involved in delivering local, national, and international health care services aimed at the treatment of esophageal cancer or to evaluate direct and indirect benefits, harms, and costs related to the various procedures used to treat the disease. This document may also serve as the basis for adaptation by local, regional, or national guideline panels.

### Description of the health problems

#### Background

The global incidence of esophageal cancer is increasing, with 2022 data recording at least 511,000 new cases worldwide with 43,571 from Europe, and 2024 data from the USA recording 22,370 new cases.^1^ Eastern populations report higher rates of squamous cell carcinoma, with adenocarcinoma more common in the Western world, both with a male preponderance.

Preoperative staging is challenging with significant limitations of available diagnostic modalities, and there remains a risk of staging misclassification. Computed tomography (CT) and endoscopic ultrasound (EUS), widely used, have limited both specificity and sensitivity for accurately describing stage data. CT is poor at detecting T stage, with an accuracy of only 40–50%. EUS is better than CT for T stage, though still struggles to differentiate T2 from T3 disease. EUS is also better than CT for detecting nodal status, particularly if cytology is assessed, though accuracy is still under 90%, and occult nodal metastasis rates of up to 50% have been reported.^2^^,^^3^ If local treatment is implemented based on under staged disease, particularly under staged nodal stage, then treatment failure is likely.

Early-stage esophageal cancers, particularly those confined to the mucosa, are often successfully treated by local resection. More advanced tumors require more aggressive treatment as lymph node involvement is common. For intermediate stage disease, such as cT2N0, accurate staging becomes of outmost importance and the need to accurately exclude nodal metastasis is paramount. Available diagnostic modalities are not foolproof and therein lies a source of controversy in management of cT2N0 disease.

European and US groups, such as ESMO and the NCCN respectively, have suggested that neoadjuvant treatment should be implemented for cT2N0M0 disease. Previous recommendations were in favor of chemoradiotherapy for all cellular types but more recent recommendations for esophageal adenocarcinoma support neoadjuvant chemotherapy without radiation (e.g. FLOT protocol).

There is data to suggest that some cT2N0 tumors may be at low risk of nodal metastases. Low risk features include well-differentiated cellular grade, small tumor size, lack of lymphovascular invasion on biopsy and other criteria, and these may be amenable to primary resection. In this scenario, confidence in staging remains of utmost importance. The Japanese Esophageal Society, which heavily relies on EUS and preoperative histology for staging, tends to recommend primary surgery for these presumed lower risk groups. If successful, avoiding chemotherapy and radiotherapy would prevent neoadjuvant therapy-related toxicity, avoid overtreatment, decrease costs, and decrease treatment times.

There has been an evidence gap related to perioperative treatment of cT2N0 esophageal cancer, with recognized trials such as the CROSS trial^4^ and the ESOPEC trial^5^ not specifically examining cT2N0 disease and only examining this subgroup with limited number on post hoc analysis. No RCT has conclusively compared surgery alone with neoadjuvant therapy for cT2N0 disease.

## METHODS

A Working Group within the ISDE Guidelines Committee performed a systematic review of the literature. This working group comprised 2 surgeons, two research fellows and a methodologist. The group encompassed expertise in the management of both esophageal squamous cell cancer and esophageal adenocarcinoma. The search was performed on 25 November 2024.

**Fig. 1:**
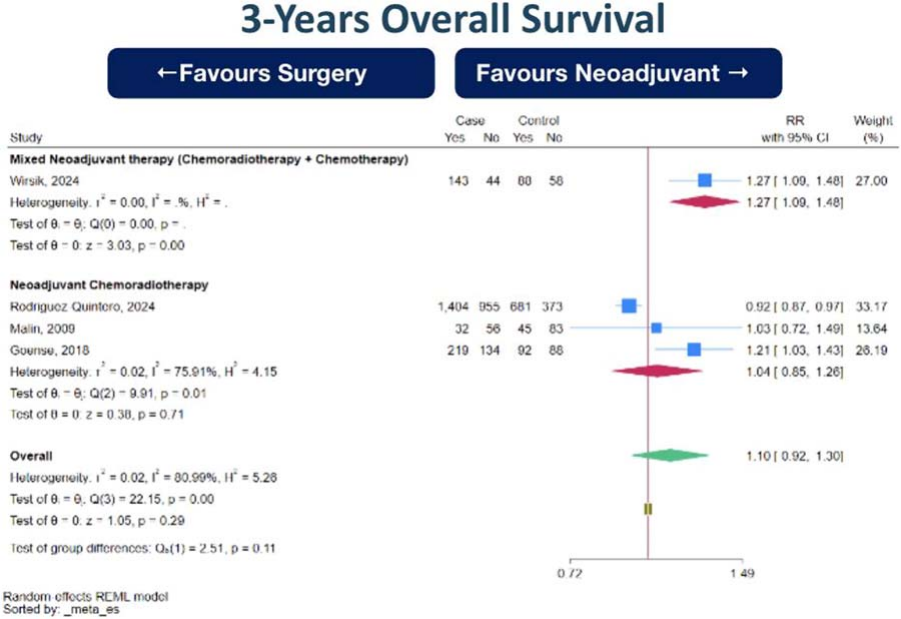
Forest plot of 3-years overall survival (OS) between neoadjuvant therapy followed by surgery (NeoAdj+S) and primary surgical resection (PSR), by neoadjuvant therapy type. Abbreviation: risk ratios, RR.

The systematic review was performed according to the Preferred Reporting Items for Systematic Review and Meta-Analysis (PRISMA) checklist^6^ (Fig. 1). Results of the systematic review were presented to a panel of experts selected by the wider Guidelines Committee, and these results informed the panel discussion about the guideline.

This panel used the Grading of Recommendations Assessment, Development and Evaluation (GRADE) approach to deliberate and formulate recommendations, in a manner similar to that reported elsewhere.^7^ Reporting of this guideline was structured as per the Essential Reporting Items for Practice Guidelines in Healthcare (RIGHT) checklist.^8^

### Guideline panel organization

International experts on esophageal cancer were invited to participate in the ISDE Guideline Panel. All panel members were experienced clinicians with training in either esophageal surgery, medical oncology or radiation oncology, and submitted disclosures on potential conflicts of interest. A non-voting patient from the Queen Elizabeth Hospital Birmingham Oesophageal Cancer Patient Support Group was also included in discussion and decision-making. A methodologist with extensive guideline development experience (MAJ) and the ISDE working group research fellows (YE, SL) participated in the panel as non-voting members and facilitated appraisal of the evidence and formulation of the recommendations. A full list of all contributors to the guideline development is provided in Appendix A.

The panel reviewed the literature presented by the fellows and met via video conference to create the recommendation. The panel reviewed evidence tables populated by the systematic review results and voted on components of Evidence tables to reach final recommendations.

### Guideline funding and declaration and management of competing interests

Funding for the methodologist was provided by Cabrini Hospital, as was a research stipend for one research fellow (YE). No industry support was used to create this guideline, nor was any industry input used for any stage of the development, dissemination, or implementation of this guideline. Standard disclosure forms from the journal Diseases of the Esophagus were completed by all guideline contributors to evaluate for potential conflict of interest. Evaluation was made by the panel Chair, and no potential conflicts were deemed to have affected the decision.

### Selection of questions and outcomes of interest

The Guidelines Update Task Force performed a literature search on management of cT2N0 esophageal cancer using the systematic review search syntax detailed in Table 1. PSR and (neoadjuvant therapy followed by surgery) were identified as comparators of interest, and key questions were formulated according to the patient-intervention-comparator outcome (PICO) format. Outcomes that were selected ‘critical’ or ‘important’ for patients were selected *a priori* as outcomes potentially critical or important for decision-making after evidence had been collected. These outcomes centered on efficacy, safety, and side effects associated with esophagectomy, chemotherapy, radiotherapy, and combined modality therapy. These outcomes included overall survival (OS), disease-free survival (DFS) rates, and complication rates.

**Table 1:** Study characteristics

				Sample size					Histology			
Study	Year	Country	Design	Total (N)	NeoAdj + S (N)	PSR (N)	Male (%)	Mean age (years)	EAC (N)	SCC (N)	Neoadjuvant therapy type	Risk of bias assessment
Capovilla	2021	Italy	Retrospective cohort	229	38	191	83.8	61.2	96	133	Chemoradiotherapy	High
Chen	2012	Taiwan (China)	Retrospective cohort	71	57	14	90.2	57.4	-	71	Chemoradiotherapy	Low
Crabtree	2013	USA	Retrospective cohort	752	270	482	-	63.8	182	29	Chemoradiotherapy + Chemotherapy + Radiotherapy	Moderate-to-high
Dolan	2016	USA	Retrospective cohort	27	11	16	93.4	65.9	25	2	Chemoradiotherapy	Moderate-to-high
Goense	2018	Netherlands	Retrospective cohort	533	353	180	74.4	64.3	422	111	Chemoradiotherapy	Low
Hardacker	2014	USA	Prospective cohort	68	33	35	80.9	61.7	57	11	Chemoradiotherapy	Moderate-to-high
Lin	2024	China	Retrospective cohort	583	316	267	78.6	-	423	160	Chemoradiotherapy	Low
Malin	2009	USA	Prospective cohort	216	88	128	-	-	-	33	Chemoradiotherapy	Moderate-to-high
Markar	2016	France	Retrospective cohort	355	70	285	80.6	-	171	184	Chemoradiotherapy + Chemotherapy	Low
Martin	2013	USA	Retrospective cohort	490	223	267	77.6	64.9	-	-	Radiotherapy	Low
PerezHolguin	2021	USA	Retrospective cohort	1300	821	479	-	-	-	-	Chemoradiotherapy	Moderate-to-high
Rhodin	2021	USA	Retrospective cohort	2540	1363	1177	82.8	64.4	-	461	Chemoradiotherapy + Chemotherapy	Moderate-to-high
Rodriguez-Quintero	2024	USA	Retrospective cohort	3413	2359	1054	88.1	64.7	3413	-	Chemoradiotherapy	Moderate-to-high
Samson	2016	USA	Retrospective cohort	1785	853	932	82.7	65.5	-	-	Chemoradiotherapy + Chemotherapy	Moderate-to-high
Song	2016	China	Retrospective cohort	243	151	92	-	-	-	-	Radiotherapy	Moderate-to-high
Speicher	2014	USA	Retrospective cohort	1559	688	871	84.0	63.8	-	-	Chemoradiotherapy + Chemotherapy + Radiotherapy	Moderate-to-high
Wirsik^*^	2024	Germany	Retrospective cohort	333	187	146	82.3	67.3	-	-	Chemoradiotherapy + Chemotherapy	Moderate-to-high
Zhang	2012	USA	Retrospective cohort	69	55	14	85.5	62.6	54	15	Chemoradiotherapy	Moderate-to-high

Abbreviations: primary surgical resection, PSR; neoadjuvant therapy followed by surgery, NeoAdj+S; esophageal adenocarcinoma, EAC; squamous cell carcinoma, SCC.

^*^Only study that used FLOT and CROSS protocols; the rest of included studies are non-FLOT, non-CROSS, or not reported.

### Evidence appraisal

Results from the ISDE systematic review and meta-analysis were presented to the Panel in the form of Forest plots and associated metrics, to facilitate evidence appraisal and panel decision-making.

Methods outlined in the GRADE handbook were used to judge the certainty of evidence for each outcome of interest. Evidence tables were created. The highest level of data available was used for the tables; less rigorous data that addressed the same outcomes was reviewed but not used in decision making. In brief, the research fellows and methodologist judged the certainty of the body of evidence across the domains of risk of bias, inconsistency, indirectness, and imprecision. The Newcastle-Ottawa Scale (NOS) was used for assessing the risk of bias of nonrandomized studies. If there was concern in any one of these domains, the certainty was downgraded.

### Assumed values and preferences

As this guideline have a patient-centered rather than a societal perspective, the panel members used their collective patient experience to make judgements about patient values and preferences. The target audience includes patients, surgeons, and oncologists.

### Development of clinical recommendations

The outcomes from the evidence table deemed critical and important to decision-making were determined by the panel. The panel discussed desirable effects, undesirable effects, the certainty of evidence, the potential variation in values of key stakeholders, balance of these effects, and acceptability and feasibility of the option favored by the balance of effects. After discussing the available evidence for each of these components, as well as pertinent additional considerations noted by the panelists based on interpretation of the evidence or expert experience, the panel would vote on each component. Finally, a vote was conducted on the final recommendation for that key question. The recommendation was made when ≥70% of cast votes were in favor.

The panel addressed subgroups such as cancer histological subtypes and grades, molecular markers, and tumor length and location within the esophagus during a discussion for the justification for their recommendation. These considerations are specified below. The evidence, additional considerations, and final judgements for each step in the decision-making process are summarized in the recommendations that follow.

### Meta-analysis

This study followed PRISMA guidelines. For categorical outcomes, proportions with confidence intervals were extracted; for continuous outcomes, means and standard deviations were used, converting medians or ranges when necessary. Pooled estimates were obtained using random-effects models with restricted maximum likelihood to account for between-study variability. Heterogeneity was assessed with Q, τ^2^ and I^2^ statistics. Publication bias was evaluated with funnel plots and tested using Egger’s and Begg’s methods, with the trim-and-fill approach applied when bias was detected. Where possible, meta-regression and subgroup analyses explored potential sources of heterogeneity. Sensitivity analyses were performed using a leave-one-out approach. All analyses were conducted in Stata (version 18.5).

### Guideline document review

This guideline was drafted based on the evidence tables created by panel voting and discussion. All panel members and working groups members then edited the guideline. The final version of the guideline was then submitted to ISDE leadership for approval before being published online for public comment for 2 weeks. After this public comment period, the final version of this guideline was submitted for final publication.

KEY QUESTION

**Should PSR OR neoadjuvant therapy followed by surgery be used for cT2N0 esophageal cancer in adults?**

**Recommendations.**

Given the limitations of staging accuracy and the absence of direct trial data, the ISDE Guideline panel suggests that

NEOADJUVANT THERAPY FOLLOWED BY SURGERY IS PREFERABLE TO PSR FOR MOST PATIENTS WITH cT2N0 ESOPHAGEAL CANCER.

*(Conditional recommendation*, *low certainty of evidence.)*

Remark: primary surgery alone may be considered in exceptional cases meeting all of the following: small, well-differentiated tumor, no LVI, and high confidence in staging workup.

No separate recommendation is made by histological subtype, as the available evidence was insufficient to show differential effects.

The evidence does not demonstrate a survival advantage for neoadjuvant therapy in cT2N0 esophageal cancer. However, the panel’s consensus was that high rates of nodal upstaging would favor the use of neoadjuvant therapy and avoid under-treatment of patients in real-world practice.

### Summary of the evidence

A total of eighteen studies were included in this analysis. To the extent possible, it was determined that no data were double-counted from any of the included large databases. No randomized controlled trials (RCTs) were identified. Fourteen were retrospective cohort studies, two were retrospective cohort studies with propensity score matching, and two were prospective cohort studies. All included studies were comparative studies between PSR and neoadjuvant therapies followed by surgery (NeoAdj+S). Among these, neoadjuvant chemoradiotherapy was used in ten studies, neoadjuvant radiotherapy alone was used in two studies, and the remaining six studies were a mixture of chemotherapy alone, radiotherapy alone, or combined modality therapy (Table 1).

Risk of bias was considered to be high among studies included in this analysis. Of the 18 included studies only five studies (28%) were rated as low risk of bias, scoring above 7 on the NOS (Table 2). The other 13 studies received a moderate-high or high risk of bias rating. In particular, studies were weakest in the Comparability domain of the NOS which assesses the degree to which studies have been controlled for confounding (Table 2).

**Table 2:** Newcastle-Ottawa scale (NOS) risk of bias assessment

Study	Design	Selection (4 items, 4 points)	Comparability (1 items, 2 points)	Outcome (3 items, 3 points)	Total risk of bias score
Capovilla 2021	Retrospective cohort study	4	0	1	5
**Chen 2012**	Retrospective cohort study	4	2	2	8
Crabtree 2013	Retrospective cohort study	4	1	1	6
Dolan 2016	Retrospective cohort study	3	1	2	6
**Goense 2018**	Retrospective cohort study	4	2	2	8
Hardacker 2014	Prospective cohort study	3	1	2	6
**Lin 2024**	Retrospective cohort study- Propensity matched	4	2	2	8
Malin 2009	Prospective cohort study	3	0	3	6
**Markar 2016**	Retrospective cohort study	4	2	2	8
**Martin 2013**	Retrospective cohort study	4	1	2	7
PerezHolguin 2021	Retrospective cohort study	4	0	2	6
Rhodin 2021	Retrospective cohort study	4	0	2	6
Rodriguez-Quintero 2024	Retrospective cohort study—propensity matched	3	1	2	6
Samson 2016	Retrospective cohort study	4	1	1	6
Song 2016	Retrospective cohort study	4	0	2	6
Speicher 2014	Retrospective cohort study	4	0	2	6
Wirsik 2024	Retrospective cohort study	4	0	2	6
Zhang 2012	Retrospective cohort study	3	1	2	6

NOS domain criteria:

Selection (4 items): (1) representativeness of the exposed cohort, (2) selection of the non-exposed cohort, (3) ascertainment of exposure, (4) demonstration that outcome of interest was not present at the start of the study. **Bolded** studies have the lowest risk of bias.

### Benefits

**Fig. 2:**
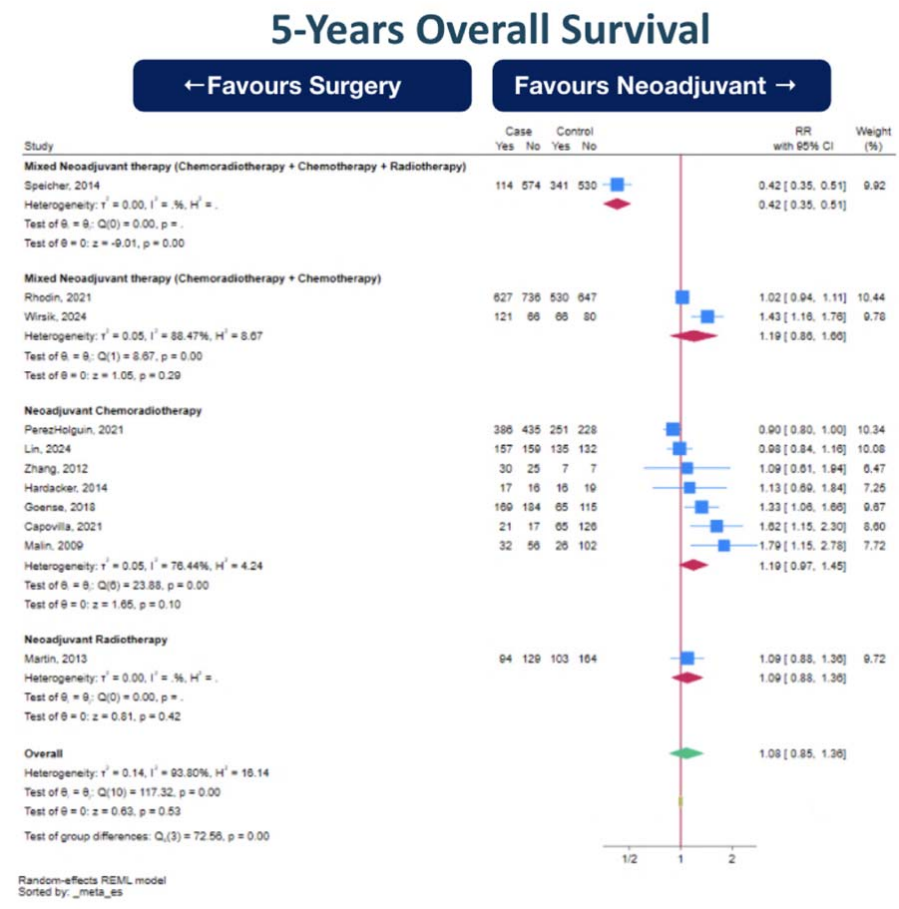
Forest plot of 5-years overall survival (OS) between neoadjuvant therapy followed by surgery (NeoAdj+S) and primary surgical resection (PSR), by neoadjuvant therapy type. Abbreviation: risk ratios, RR.

**Fig. 3:**
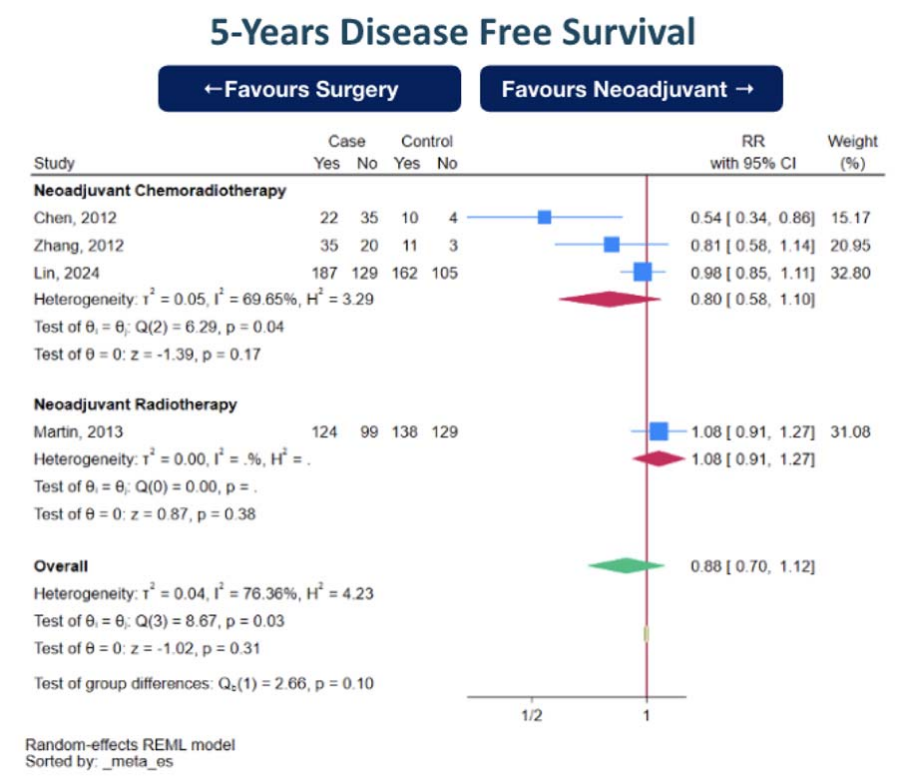
Forest plot of 5 disease-free survival (DFS) between neoadjuvant therapy followed by surgery (NeoAdj+S) and primary surgical resection (PSR), by neoadjuvant therapy type. Abbreviation: risk ratios, RR.

**Fig. 4:**
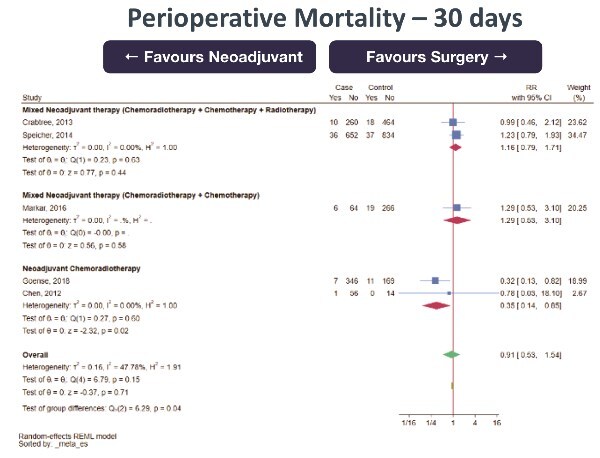
Forest plot of 30-day mortality between neoadjuvant therapy followed by surgery (NeoAdj+S) and primary surgical resection (PSR), by neoadjuvant therapy type. Abbreviation: risk ratios, RR.

**Fig. 5:**
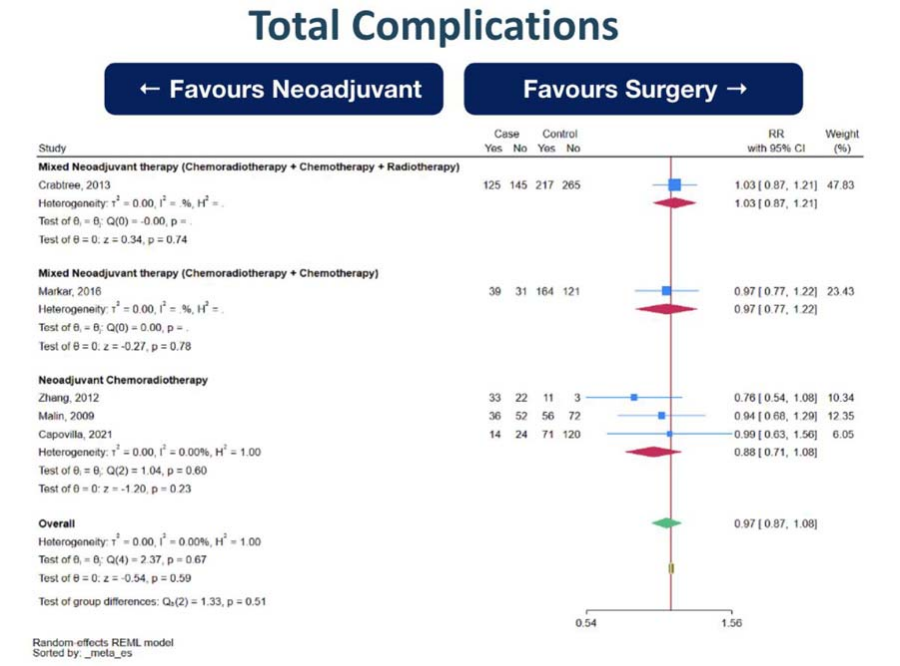
Forest plot of total complications between neoadjuvant therapy followed by surgery (NeoAdj+S) and primary surgical resection (PSR), by neoadjuvant therapy type. Abbreviation: risk ratios, RR.

PSR had similar effectiveness as neoadjuvant therapy (all combinations) followed by surgery (NeoAdj+S) with regards to:

- 3-year OS (0 RCT, 1 prospective cohort study, 3 retrospective cohort studies; total 4495 subjects, very low certainty of evidence, RR 1.10 [95%CI 0.92–1.30] favoring NeoAdj+S) with high heterogeneity (Fig. 1)
- 5-yearr OS (0 RCT, 2 prospective cohort studies, 9 retrospective cohort studies; total 7920 subjects, very low certainty of evidence, RR 1.08 [95%CI 0.85–1.36] favoring NeoAdj+S) with high heterogeneity (Fig. 2).
- 5-year DFS (0 RCT, 4 retrospective cohort studies; total 1213 subjects, very low certainty of evidence, RR 0.88 [95%CI 0.70–1.12] favoring PSR) with high heterogeneity (Fig. 3)

The combined magnitude of these effects was thought by the panelists to be small. Additional supplementary figures may be found in supplementary Appendix B.

### Harms and burden

**Fig. 6:**
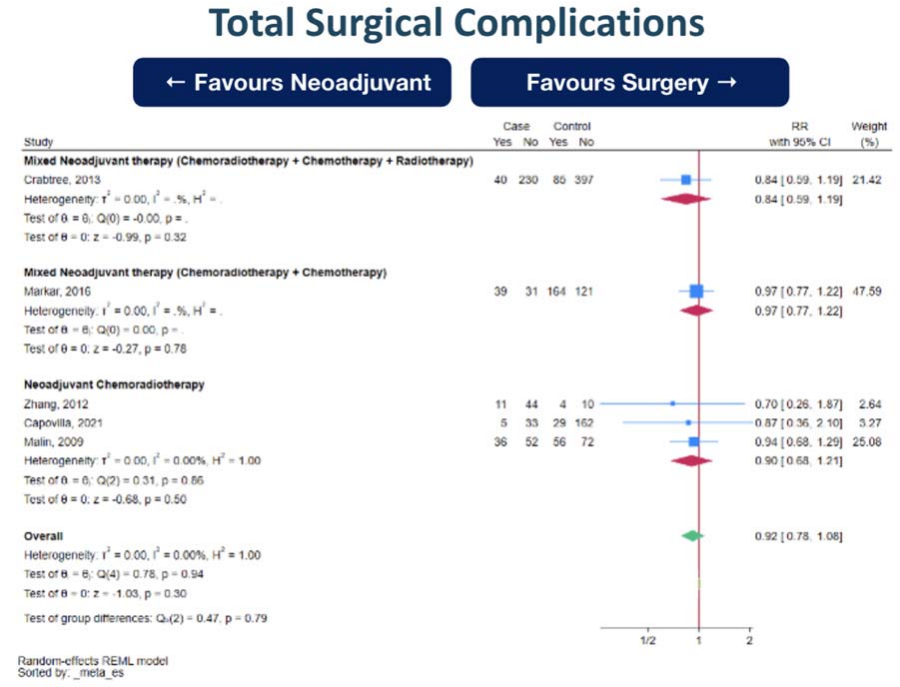
Forest plot of surgical complications between neoadjuvant therapy followed by surgery (NeoAdj+S) and primary surgical resection (PSR), by neoadjuvant therapy type. Abbreviation: risk ratios, RR.

**Fig. 7:**
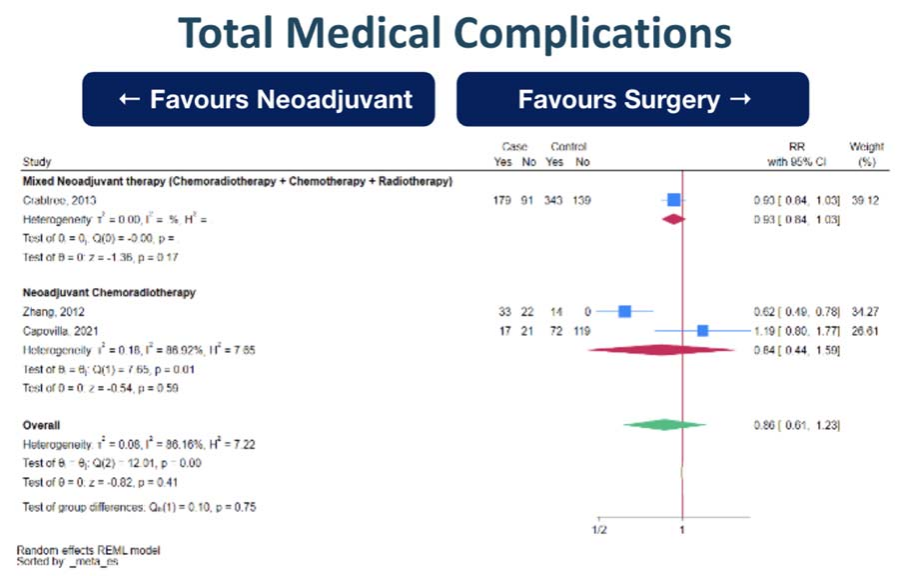
Forest plot of medical complications between neoadjuvant therapy followed by surgery (NeoAdj+S) and primary surgical resection (PSR), by neoadjuvant therapy type. Abbreviations: risk ratios, RR.

PSR had similar harms as neoadjuvant therapy (all combinations) followed by surgery (NeoAdj+S) with regards to:

- 30-day mortality (0 RCT, 5 retrospective cohort studies; total 3270 subjects, very low certainty of evidence, RR 0.91 [95%CI 0.53–1.54] favoring NeoAdj+S) with moderate heterogeneity (Fig. 4)
- Total complications (0 RCT, 1 prospective cohort studies, 4 retrospective cohort studies; total 1621 subjects, very low certainty of evidence, RR 0.97 [95%CI 0.87–1.08] favoring NeoAdj+S) with low heterogeneity (Fig. 5)
- Surgical complications (0 RCT, 1 prospective cohort study, 4 retrospective cohort studies; total 1621 subjects, very low certainty of evidence, RR 0.92 [95%CI 0.78–1.08] favoring NeoAdj+S) with low heterogeneity (Fig. 6)
- Medical complications (0 RCT, 3 retrospective cohort studies; total 1050 subjects, very low certainty of evidence, RR 0.86 [95%CI 0.61–1.23] favoring NeoAdj+S) with high heterogeneity (Fig. 7)

The combined magnitude of these effects was thought by the panelists to be small. Additional supplementary figures may be found in Appendix B.

### Certainty of evidence

The certainty in these effects was rated as very low owing to the risk of bias and imprecision of the estimates. The overall certainty of evidence was deemed very low.

### Decision criteria and additional considerations

The panel considered the value for decision-making that well-informed patients would place on the main outcomes based on their experience and the available evidence. Direct patient input was also incorporated in this discussion regarding the assignment of relative values of outcomes.

Longest OS data was deemed by the panel as the most critical outcome, though the patient advocate ranked this second to 30-day mortality, with other complications being ranked less important by both panel and patient. Our analysis found that OS, 30-day mortality rates, and total complication rates were largely equivalent in both PSR patients and NeoAdj+S patients.

The panel acknowledged the results of the meta-analysis. However, the poor accuracy of preoperative clinical staging was of concern, particularly understaging of nodal disease, as knowledge of lymph node involvement would strongly support neoadjuvant therapy before operation. Also, certain biological features of the tumor, such as poorer differentiation, large size and lymphovascular invasion would tend to direct experts in favor of NeoAdj+S, irrespective of actual clinical TNM staging.

Accuracy of preoperative clinical staging, that is the correlation of cTNM with pTNM, was noted to be poor, even in the modern era with accessibility of EUS, MRI, flexible bronchoscopy, and PET scans. Accurate staging was reported in approximately 6–42.8% of cases across the reviewed studies.^9^^,^^10^

Upstaging after histopathological review of the resected specimen was common (range 21.4–63.4% of cases)^11^ (Table 3). Most concerningly, among all patients presumed to be cT2N0M0, upstaging occurred secondary to unexpected nodal disease in up to 48% of cases, highlighting limitations in preoperative nodal assessment. The panel agreed that PSR of the tumor, but leaving lymph nodes involved with tumor as would be seen when node-negative disease is unexpectedly upstaged to node-positive disease, would predictably lead to recurrent disease. The frequency of postoperative adjuvant treatment for pathologically upstaged tumors was unclear in some studies, introducing a confounder into the analysis.

**Table 3:** Correlation between clinical staging of cT2N0M0 esophageal cancers and post-resection pathological staging

Study	Year	Staging modality	Percentage of patients correctly staged	Percentage of total patients upstaged	Percentage of total patients upstaged due to change in nodal status
Capovilla	2021	CT, EUS, PET, Bronchoscopy	21.4%	63.4%	26%
Chen	2012	CT, EUS, Bone scan, Barium swallow	42.8%	25.0%	7.0%
Crabtree	2013	No reported	27.4%	46.7%	38.2%
Dolan	2016	CT, EUS, Endoscopy, ± PET	6%	56%	56%
Goense	2018	CT, EUS, ± PET	38%	62%	45%
Hardacker	2014	CT, EUS	8.5%	48.5%	40%
Malin	2009	CT, Endoscopy, Barium swallow ± EUS/PET ± bronchoscopy	-	-	-
Markar	2016	CT, EUS, ± PET	-	-	48.1%
PerezHolguin	2021	Not reported	30.1%	28.7%	21.3%
Rodriguez-Quintero	2024	Not reported	-	-	35.3%
Samson	2016	Not reported	-	45.7%	30.1%
Speicher	2014	Not reported	26.7%	41.6%	30.2%
Wirsik	2024	CT, Endoscopy ± PET± EUS	-	-	-
Zhang	2012	CT ± EUS	28.6%	21.4%	16.05%

Abbreviations: computed tomography, CT; endoscopic ultrasound, EUS; positron emission tomography, PET.

Poor differentiation of the primary cancer was thought by the panel to be a factor suggesting the need for a NeoAdj+S approach, and this is reflected in current AJCC 8th edition staging for esophageal cancer, wherein T2N0M0 adenocarcinoma is upstaged from Stage IC to IIA in the presence of poor differentiation, and squamous cell cancer is upstaged from Stage IB to IIA unless the cells are well-differentiated. Beyond poor differentiation, the panel agreed that size and lymphovascular invasion are additional high-risk features. The panel considered tumors ≥2 cm in length to be considered large.

The panel recognizes that cancer patterns differ between geographical locations, with a panel member from Japan emphasizing the higher percentage of squamous cancer in the Japanese population. Moreover, there is greater patient awareness of the disease in Japan, and some well-informed patients may request organ preservation techniques with definitive chemoradiation even for cT2N0 disease.^12^

There were also subgroups of patients for whom the panel members expressed a preference for PSR, such as in elderly patients deemed unfit for neoadjuvant therapy based on physiological parameters. The corollary held true, in that the panel confirmed their preference for NeoAdj+S for younger patients, particularly those thought fit enough by physiological metrics to be able to tolerate FLOT protocol chemotherapy. However, even in this group, shared decision making with the patient is of utmost importance, because in a very selected subgroup of young healthy patients, with well-differentiated cancers, no high-risk features (such as lymphovascular invasion), and a very small tumor (suggested by the panel to be defined as <2 cm in length), some experts would consider PSR as a preferable option. When deciding upon a PSR approach, preoperative staging should utilize best available modalities, thought by the panel to be routine FDG-PET and EUS.

For the intermediate group, those patients (particularly with adenocarcinoma) thought unable to tolerate full course neoadjuvant FLOT chemotherapy, there was some preference for the less effective, but better tolerated CROSS protocol chemoradiation. Specific physiologic criteria were not able to be agreed upon, and the importance of multidisciplinary review by surgeons, oncologists, and radiologists, in conjunction with the patient, was stressed.

Finally, the panel agreed that a preferred comparative study would be clinical staging with all available modalities, matched for high-risk features of size, grade, and lymphovascular invasion, and then comparing the neoadjuvant FLOT protocol chemotherapy with CROSS chemoradiation, and both against primary resection. Emerging data reviewing cancers deficient in DNA mismatch repair (dMMR) and its effect on choice of treatment were discussed without definitive recommendations being formulated. Subgroup analysis was considered in this guideline, particular examining histological subtype, but comparative data was minimal and clear description of protocols limited in the included publications, and therefore this analysis was not performed.

It was re-emphasized by the panel that there was evidence to support some of the above findings, while other findings lacked evidence and relied heavily on expert opinion (Table 4).

**Table 4:** Summary of evidence-based results and expert consensus based beliefs

Summary of conclusions drawn from evidence	Summary of expert consensus beliefs drawn from clinical experience
PSR and NeoAdj+S are **similarly effective** with regards to **5**-**year overall survival** (very low certainty of evidence) PSR and NeoAdj+S are **similarly effective** with regards to **3**-**year overall survival** (very low certainty of evidence) PSR and NeoAdj+S are **similarly effective** with regards to **5**-**year disease-free survival** (very low certainty of evidence) PSR and NeoAdj+S have **similar harms** with regards to **30**-**day mortality** (very low certainty of evidence) PSR and NeoAdj+S have **similar harms** with regards to **total complications** (very low certainty of evidence) PSR and NeoAdj+S have **similar harms** with regards to surgical complications (very low certainty of evidence) PSR and NeoAdj+S have **similar harms** with regards to **medical complications** (very low certainty of evidence) Accuracy of preoperative clinical staging was heterogenous between studies included in the meta-analysis (reported as 6–42.8% of cases accurately staged), with use of varied staging modalities Upstaging was reported in several of the studies included in the meta-analysis, and was reported to occur in 21.4–63.4% of patients Upstaging secondary to unexpected nodal disease was reported in several of the studies included in the meta-analysis, and was reported to occur in 7.0–48.1% of patients	Longest overall survival is the most critical outcome for patients Modalities that should routinely be utilized to stage esophageal cancer include both FDG-PET and endoscopic ultrasound Understaging of esophageal cancer as cT1N0M0 is likely to result in disease recurrence, particularly if the nodal status is understaged In a patient that is physiologically fit or has additional pathological risk factors, neoadjuvant treatment is preferable due to the risk of incorrect clinical staging Physiological fitness must be determined in a multidisciplinary setting Additional high risk pathological features include poor differentiation of primary cancer, larger tumor size, presence of lymphovascular invasion, tumor length 33 cm

### Values

The panel voted that there was certainty that OS from esophageal cancer was the most important outcome of treatment of cT2N0 disease, with 75% of the panel voting that there was either ‘probably no important uncertainty’ or only ‘possible uncertainty’ in how much patients might value the main outcome. DFS, quality of life issues, and perioperative complications were thought to be less important. However, the patient representative was most concerned with perioperative complications, in particular perioperative mortality, though the representative felt that the list of outcomes was comprehensive, and representative of what patients care about.

### Balance of effects

The balance between desirable and undesirable effects in the data reviewed does not favor either PSR or NeoAdj+S.

### Acceptability

The panel voted that NeoAdj+S is an acceptable intervention for patients and clinicians.

### Feasibility

The panel voted that NeoAdj+S is feasible to implement relative to PSR.

### Conclusion

The panel suggests, even though pooled data from the metanalysis seem to demonstrate equivalence between PSR and NeoAdj+S for cT2N0 esophageal cancer, and based on concerns related to accuracy of clinical staging and inadequacy of defining higher risk groups, that NeoAdj+S should be preferred as a conditional recommendation and subject to shared decision making with the patient.

This decision making will consider the absence of strong data supporting this position, as well as the difference in values of the panel and the patient representative around prioritizing OS versus minimizing perioperative complications, and particularly the patients’ views on organ preservation and their acceptance of neoadjuvant therapy in general. Subgroups, particularly those patients deemed unlikely to tolerate neoadjuvant treatment protocols, may reasonably be offered primary resection. Occasionally, certain groups deemed to be of lower risk of undiagnosed nodal disease, such as patients with no high-risk features and tumors of short length, may reasonably be offered PSR in an effort to avoid potential ineffective neoadjuvant therapy. Definitive chemoradiation should be reserved only for those patients unfit for resection.

With the high risk of bias arising from the multiple potential confounding factors, any actual difference in effect between the two treatment strategies may have been masked, and the conditional recommendation was made despite these biases, driven by clinical reasoning and of the inherent risk of understaging of thereby of undertreatment.

### Subgroup considerations

- Staging factors related to poor accuracy of clinical staging methods.
- High-risk tumor factors such as tumor grade, disease length, lymphovascular invasion, dMMR status.^13^
- Older patients and those with poor physiological parameters.
- Younger, fitter patients with no high-risk factors.

### Research recommendations

The panel made several recommendations for future research considerations:

- To refine our recommendations, prospective studies are needed focusing on cT2N0 in high risk subgroups, including poorly differentiated tumors or tumors with lymphovascular invasion, as well as length of tumor.
- Explore biomarkers such as circulating tumor DNA, PD-L1^14^ in the context of staging of cT2N0 cancers.
- No specific data on neoadjuvant immunotherapy exist yet for this population, but emerging trials should assess its role,^15^ such as for patients with cancers deficient in dMMR.

### Cost analysis studies

No data was available to make a recommendation, but cost-effectiveness might be particularly relevant if intensive neoadjuvant regimens (such as FLOT or chemoradiation) are used without a clear survival benefit.

## IMPLEMENTATION OF THESE GUIDELINES

In order to implement neoadjuvant therapies prior to esophagectomy, it is necessary for facilities to have adequate infrastructure and clinician training. It is thought likely that the majority of centers capable of performing esophagectomy would have the necessary facilities and expertise to provide all forms of neoadjuvant therapy. There will be some centers, mainly in resource-poor locations of low- and middle-income countries where certain treatment options, such as radiotherapy facilities, are lacking. In such situations, weighing the benefits of local treatment versus transfer to a larger center will need to be considered.

In addition to local institutional resources, health equity considerations such as third-party payer denials (e.g. in the preoperative prescription of immune checkpoint inhibitors) may also be a barrier to implementation and should be considered by the clinician and facility managing esophageal cancers.

## MONITORING AND EVALUATION

The panel agreed that detailed clinical staging investigations with at least FDG-PET and EUS should be performed before initiating treatment by either PSR or NeoAdj+S.

Facilities performing esophagectomy should be clear about which patient populations are being selected for the respective techniques, and their comorbidities.

Tumor characteristics such as lymphovascular invasion, tumor length, MMR status, and tumor grade should be monitored. It is also suggested to monitor clinical findings and investigations which are used to evaluate the physiological suitability for various treatment regimens. Quality of life should also be measured using validated instruments at follow-up appointments.

## UPDATING THESE GUIDELINES

The ISDE Guidelines Committee plans to re-evaluate these guidelines three years post-publication.

## LIMITATIONS OF THESE GUIDELINES

The literature search strategy was limited to English language publications which introduced the risk of language bias, though with the global span of included from Europe, the Americas, and Asia, this is probably minimal. The limitations of these guidelines are inherent to the very low certainty of the evidence for the Key Question. The recommendations are thus to large extent restricted to the level of expert opinion. Furthermore, data is limited by known challenges with the accuracy of preoperative clinical staging for cT2N0 esophageal cancer. Additionally, factors which might preoperatively better determine the risk of undiagnosed nodal disease are not well understood.

Risk of Bias assessment with the NOS showed greatest weakness in the Comparability domain, with many studies lacking adjustment for prognostic factors. In particular, patients selected for primary surgery (PSR) versus neoadjuvant therapy (NeoAdj+S) may have differed systematically (e.g. tumor biology or fitness), potentially biasing outcomes. Additionally, analysis of patients upstaged by pathologic nodal status (pN+), and who subsequently received adjuvant treatment, might dilute any survival difference and confound results.

## Supplementary Material

Supplementary_material_doag019

## Abbreviations

AJCC, American Joint Committee on Cancer; ESMO, European Society for Medical Oncology; NCCN, National Comprehensive Cancer Network; T2 disease, Tumor invades muscularis propria; N0 disease, No regional lymph node metastasis; OS, Overall survival; DFS, Disease-Free survival; PSR, Primary surgical resection; NeoAdj+S, Neoadjuvant therapy followed by surgical resection; CT, Computed tomography; EUS, Endoscopic Ultrasound.

## Notes

Financial Support: This study received no funding.
