# Supplementary material for: ISDE guidelines on the management of cT2N0 esophageal cancer
Source: Dis Esophagus. 2026 Mar 5;39(2):doag019. doi: 10.1093/dote/doag019 (PMC13017695; doi:10.1093/dote/doag019)
Supplement: Supplementary_material_doag019 [file supplementary_material_doag019.zip › APPENDIX A.docx]

## APPENDIX A:

List of contributors

Panelists:

- Upper GI Surgeons: Geoffrey P Kohn, Andrew Davies, Tom Mala, Javed Sultan, Bjoern PL Wijnhoven, Ewen A Griffiths
- Thoracic Surgeons: Rudy Lackner, Nicolas Contreras, Stephanie Worrell, Virginia Litle
- Medical Oncologists: Ben Markman, Sarbajit Mukherjee, Christopher Nevala-Plagemann, Shun Yamamoto
- Radiation Oncologists: Kimberley Mak
- Methodologist: Mohammad Asghari
- Research Fellows: Yousif Eliya, Samantha Leng

Patient representative: Kam Majevadia

Research librarian: Diane Horrigan
