# Supplementary material for: ISDE guidelines on the management of cT2N0 esophageal cancer
Source: Dis Esophagus. 2026 Mar 5;39(2):doag019. doi: 10.1093/dote/doag019 (PMC13017695; doi:10.1093/dote/doag019)
Supplement: Supplementary_material_doag019 [file supplementary_material_doag019.zip › APPENDIX B.pdf]

APPENDIX B:

Supplementary Forest plots of included studies

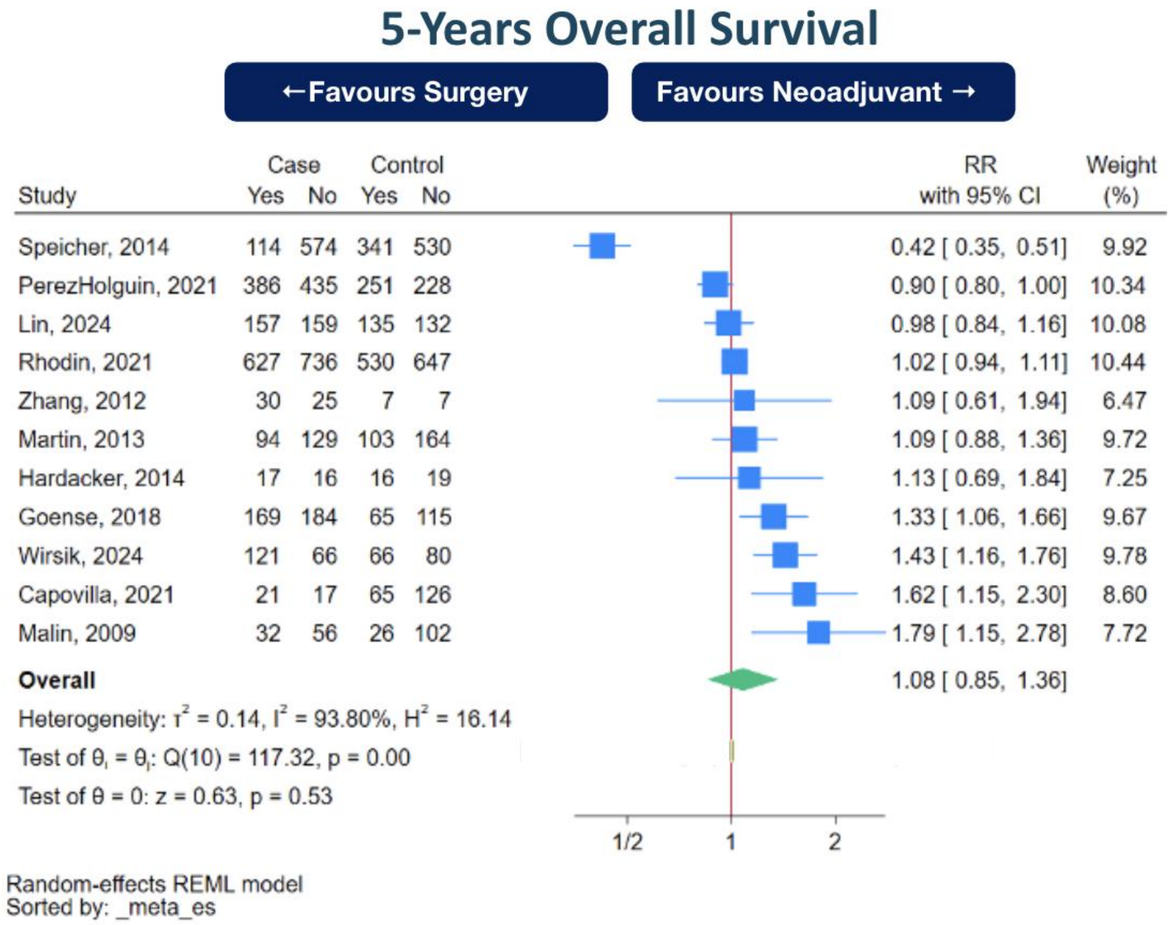

Figure 1C. Forest plot of 5-Year Overall Survival (OS) between Neoadjuvant therapy followed by surgery (NeoAdj+S) and Primary surgical resection (PSR).

Abbreviations: Risk ratios (RR).

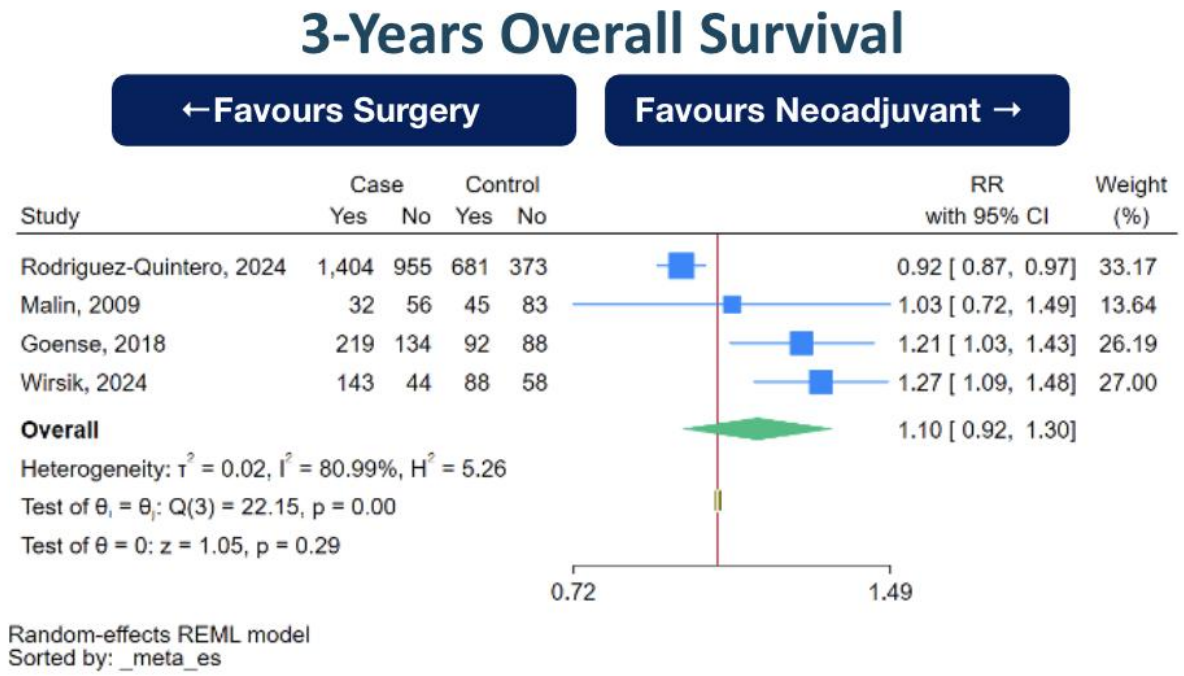

Figure 2C. Forest plot of 3-Year Overall Survival (OS) between Neoadjuvant therapy followed by surgery (NeoAdj+S) and Primary surgical resection (PSR).

Abbreviations: Risk ratios (RR).

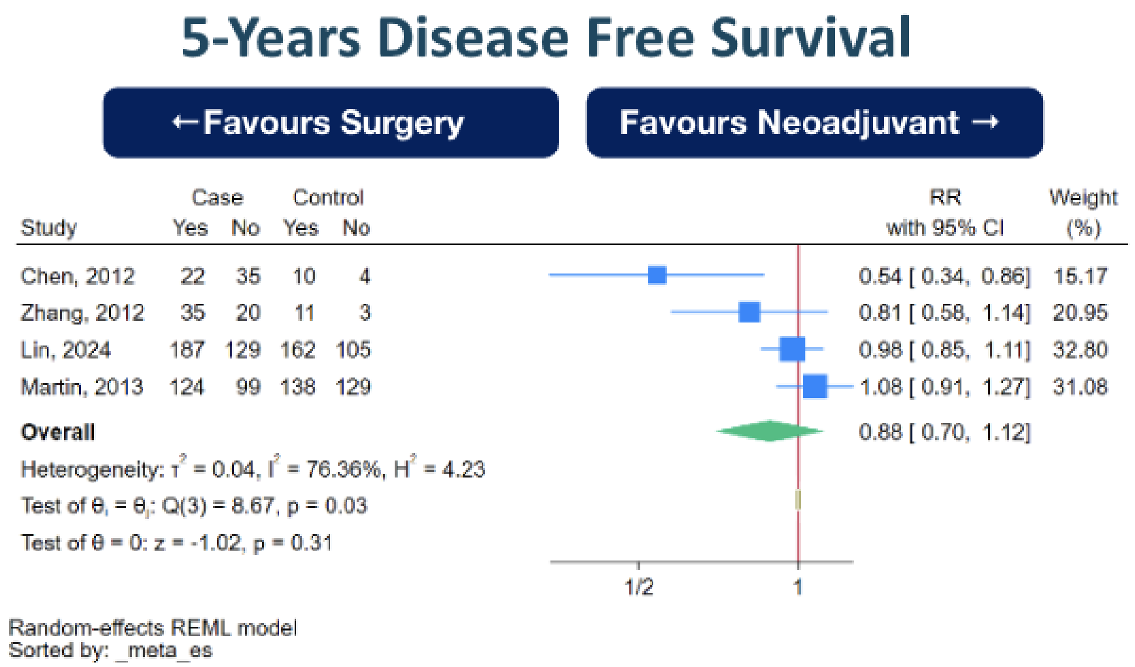

Figure 3C. Forest plot of 5yr -Disease Free Survival (DFS) between Neoadjuvant therapy followed by surgery (NeoAdj+S) and Primary surgical resection (PSR).

Abbreviations: Risk ratios (RR).

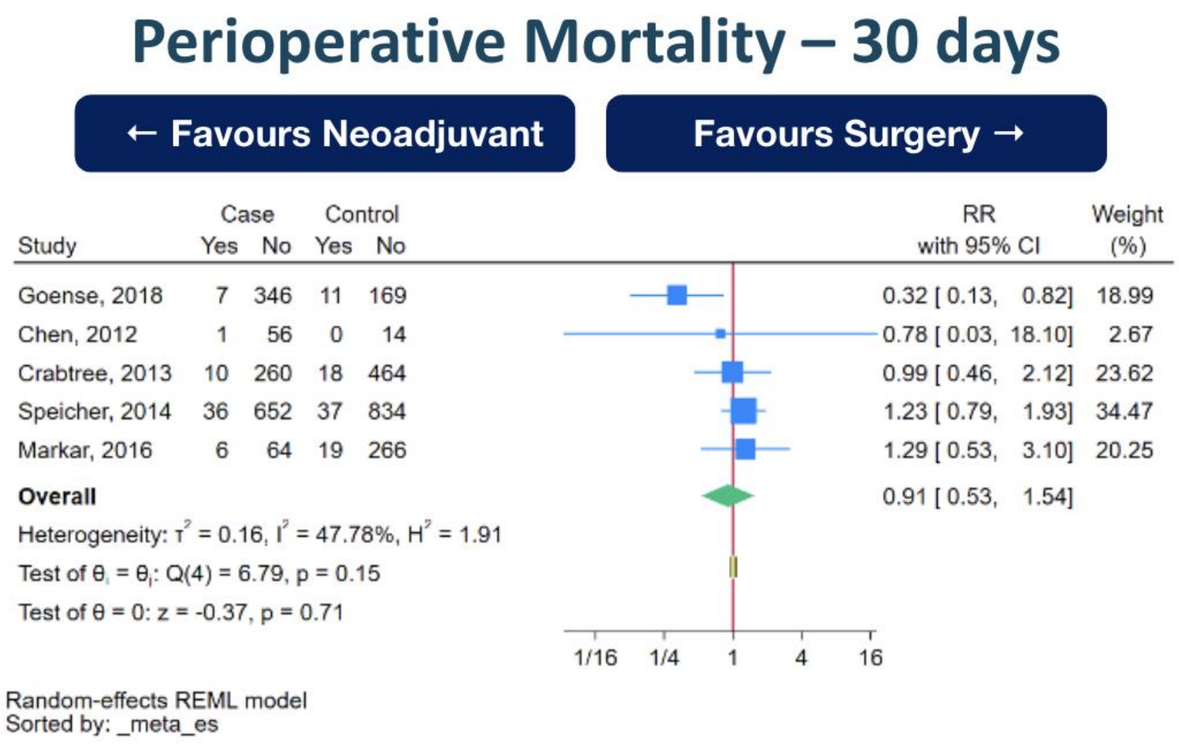

Figure 4C. Forest plot of 30-day mortality between Neoadjuvant therapy followed by surgery (NeoAdj+S) and Primary surgical resection (PSR).

Abbreviations: Risk ratios (RR).

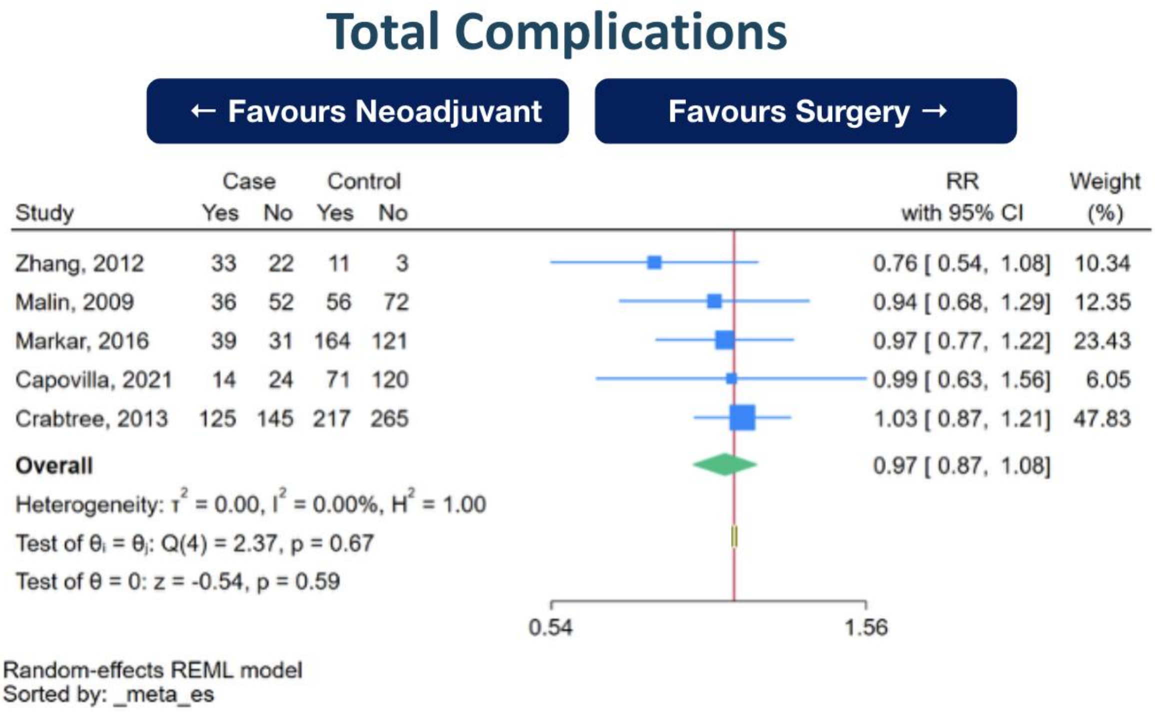

Figure 5C. Forest plot of total complications between Neoadjuvant therapy followed by surgery (NeoAdj+S) and Primary surgical resection (PSR).

Abbreviations: Risk ratios (RR).

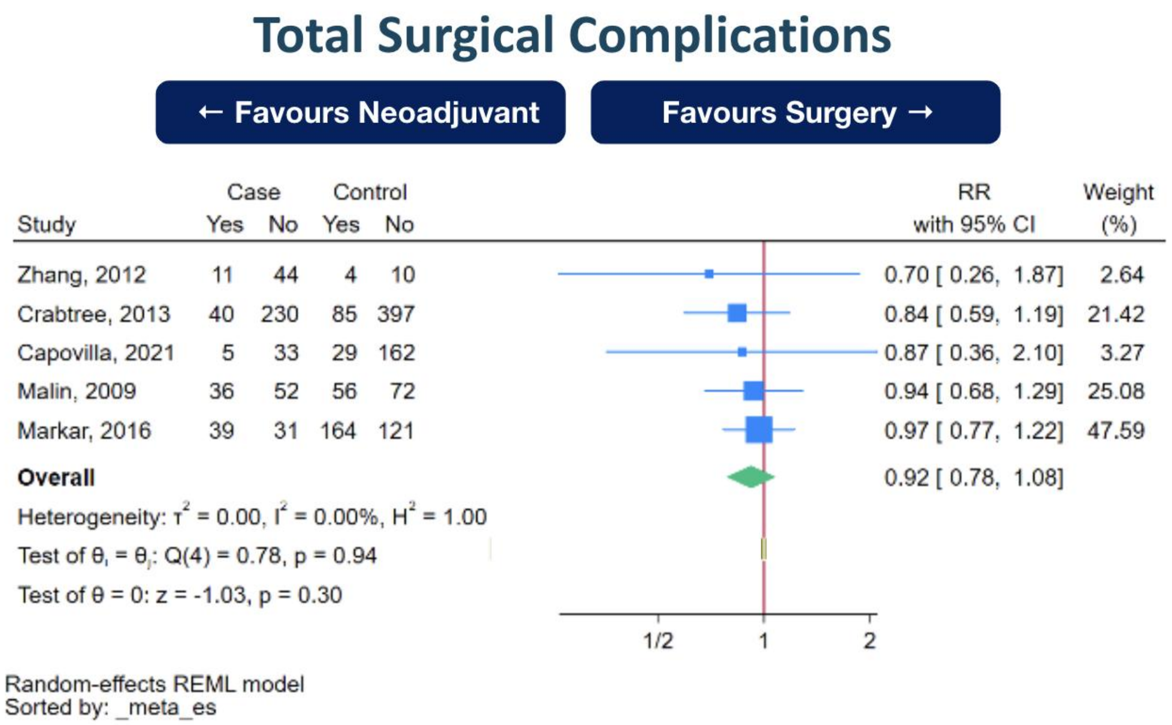

Figure 6C. Forest plot of surgical complications between Neoadjuvant therapy followed by surgery (NeoAdj+S) and Primary surgical resection (PSR).

Abbreviations: Risk ratios (RR).

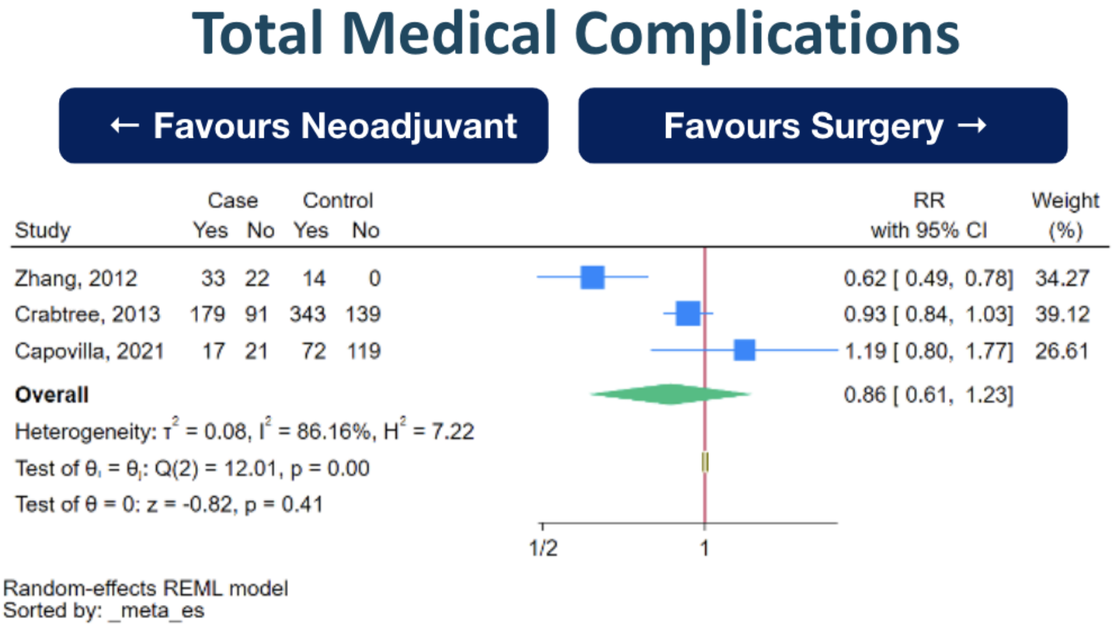

Figure 7C. Forest plot of medical complications between Neoadjuvant therapy followed by surgery (NeoAdj+S) and Primary surgical resection (PSR).

Abbreviations: Risk ratios (RR).

PRISMA chart of the included studies.

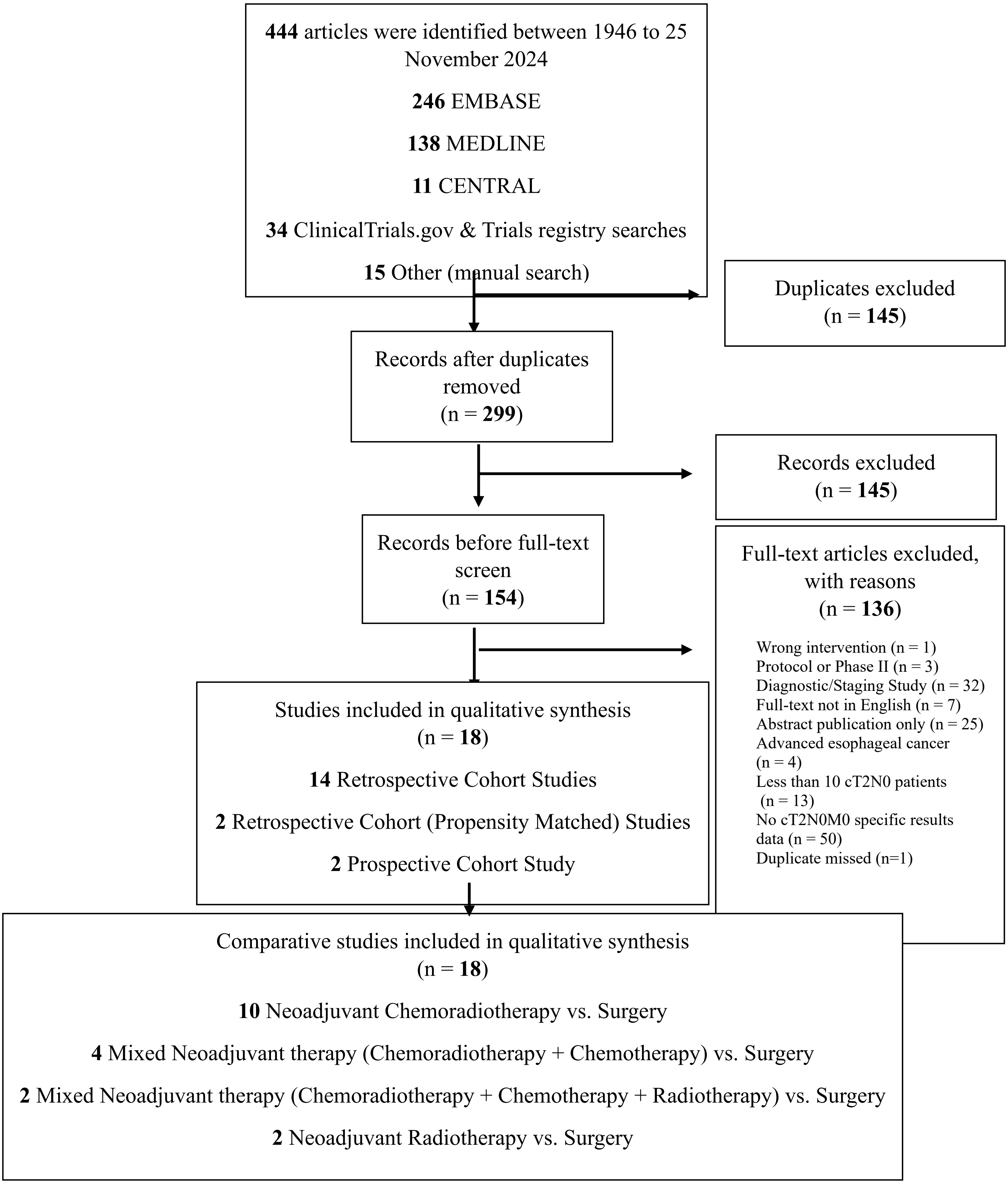

Search strategy.

MEDLINE<br>From 1946 to 25 November 2024		EMBASE<br>From 1947 to 25 November 2024		CENTRAL<br>From 1946 to 25 November 2024		ClinicalTrials.gov<br>On 25 November 2024	
#		#		#		#	
1	exp esophageal neoplasms/ (62262)	1	exp esophagus tumor/ (117103)	1	exp esophageal neoplasms/ (2674)	1	Esophageal T2N0 carcinoma
2	(oesophag* adj5 (neoplas* or cancer* or carcin* or tumo* or malig*)).tw. (9779)	2	(oesophag* adj5 (neoplas* or cancer* or carcin* or tumo* or malig*)).tw. (16305)	2	(oesophag* adj5 (neoplas* or cancer* or carcin* or tumo* or malig*)).tw. (1147)	2	Osophageal T2N0 carcinoma
3	(esophag* adj5 (neoplas* or cancer* or carcin* or tumo* or malig*)).tw. (58495)	3	(esophag* adj5 (neoplas* or cancer* or carcin* or tumo* or malig*)).tw. (84928)	3	(esophag* adj5 (neoplas* or cancer* or carcin* or tumo* or malig*)).tw. (5243)	3	Esophageal cancer
4	(adenocarcinoma$ adj5 esophag$).tw. (8894)	4	(adenocarcinoma$ adj5 esophag$).tw. (14693)	4	(adenocarcinoma$ adj5 esophag$).tw. (772)	4	Esophagogastric junction
5	Esophag* squamous cell carcinoma$.mp. (14910)	5	Esophag* squamous cell carcinoma$.mp. (26493)	5	Esophag* squamous cell carcinoma$.mp. (1151)		
6	exp esophagogastric junction/ (10802)	6	or/1-5 (139931)	6	exp esophagogastric junction/ (727)		
7	or/1-6 (93029)	7	"T2N0".ti,ab. (1331)	7	or/1-6 (7612)		
8	"T2N0".ti,ab. (742)	8	"T2 N0".mp. (1021)	8	"T2N0".ti,ab. (98)		
9	"T2 N0".mp. (616)	9	"T2 Stage".mp. (978)	9	"T2 N0".mp. (114)		
10	"T2 Stage".mp. (589)	10	t2 n0 m0.mp. (318)	10	"T2 Stage".mp. (24)		
11	t2 n0 m0.mp. (198)	11	ct2 n0 m0.mp. (15)	11	t2 n0 m0.mp. (25)		

12	ct2 n0 m0.mp. (10)	12	ct2n0.mp. (218)	12	ct2 n0 m0.mp. (2)		
13	ct2n0.mp. (113)	13	ct2n0m0.mp. (266)	13	ct2n0.mp. (11)		
14	ct2n0m0.mp. (160)	14	t2n0.mp. (1334)	14	ct2n0m0.mp. (6)		
15	t2n0.mp. (744)	15	or/7-14 (3713)	15	t2n0.mp. (102)		
16	or/8-15 (2170)	16	6 and 15 (**246 studies**)	16	or/8-15 (254)		
17	7 and 16 (**138 studies**)		--	17	7 and 16 (**11 studies**)		
